# Epidemiology of pertussis among adolescents, adults, and older adults in selected countries of Latin American: a systematic review

**DOI:** 10.1080/21645515.2020.1827613

**Published:** 2021-03-18

**Authors:** Altacilio Nunes, Ariane Abreu, Bárbara Furtado, Anderson Soares da Silva, Eduardo B. Coelho, Eliana NC de Barros

**Affiliations:** a Ribeirão Preto Medical School, University of São Paulo, Brazil; b Shift Gestão De Serviços, Rio De Janeiro, RJ, Brazil; c GSK, Rio De Janeiro, RJ, Brazil

**Keywords:** Adult, adolescent, Latin America, epidemiology, whooping cough

## Abstract

We aimed to describe the impact of pertussis on adolescents, adults, and older adults over 2007–2018 in selected Latin American countries by reviewing the literature. We searched the Medline, Embase, Scopus, LILACS, Scielo, Google Scholar, CAPES Journals Web-portal, and Cochrane databases for observational epidemiological studies, clinical trials, and systematic reviews of primary studies. Data were extracted and analyzed for all individuals aged ≥10 years. Of 6,891 studies identified only 25 were eligible. Studies were conducted in Brazil (14), Argentina (4), Colombia (4), Mexico (2) and Chile (1). Epidemiological data among target population were limited. No studies clearly assessed the status of asymptomatic or oligosymptomatic *B. pertussis* carriers in these age groups. Among all pertussis cases identified, the percentage of patients ≥10 years-old ranged between 2.1% and 66.7% depending on country and sample characteristics. The definition of cases, diagnostic methods, and age groups were not consistent across studies.

## Focus on patient section

### What is the context?

- Pertussis (whooping cough; *Bordetella pertussis*) is a vaccine-preventable, highly infectious disease transmitted rapidly through coughing, sneezing, and speaking.
- Although considered a childhood disease, pertussis is increasingly recognized as an important cause of infection and respiratory disease in adolescents, adults, and older adults.
- Diagnosing adolescents and adults with pertussis is however challenging due to asymptomatic clinical presentation and lack of sensitive diagnostic tools.
- Adolescents and adults may be carriers of the *B pertussis* pathogen and may pass on the pathogen to unimmunized or partially immunized naïve newborns and children, the population most at risk for complications and death

### What is new?

- This review consolidates what is known about the distribution of the pertussis disease among adolescents and adults in some Latin American countries, filling the knowledge gap of the epidemiology of the disease in this population.

- Diagnostic tools need to be standardized and surveillance systems need to be improved to more accurately estimate the burden of pertussis in Latin America.
- Prevention strategies such as vaccination could be applied in adolescents and adults at risk as a prevention measure.

### What is the impact?

- Epidemiological evidence is essential to assess the health risk of pertussis among pregnant women, adolescents and adults, such as healthcare professionals working with childcare, also to monitor its impact among the most vulnerable populations (newborns and children).

- This is key in defining effective health service needs and strategies, including vaccines that can prevent pertussis.

### Introduction

Pertussis (whooping cough; *Bordetella pertussis*) is a vaccine-preventable, highly infectious disease transmitted rapidly through coughing, sneezing, and speaking.^1–3^ Infection with the human-restricted gram-negative bacterium *B. pertussis* is initiated by the binding of the bacterium to tracheal and nasopharyngeal epithelium.^1,2^

The incubation period is between 7 to 10 days^1,3^ and can last up to four weeks in some patients.^3^ Pertussis generally develops in three phases:^2,3^ 1) a catarrhal phase lasting one to two weeks,^2,4^ with mild respiratory symptoms, progressing to a gradual increase in cough; 2) a paroxysmal stage,^1,2,4^ lasting 2 to 10 weeks, evolving under normal temperature and occasionally low fever, with paroxysms of dry cough, difficulty in breathing, often associated with cyanosis and apnea in children <1 year, which can lead to many complications and even death; and 3) a convalescence phase, characterized by a gradual decrease in the frequency, duration, and severity of cough that persists two to six weeks, and may extend to months.^1,3,4^

Although pertussis has mainly been considered a childhood disease, currently it is recognized as an important cause of infection and respiratory disease in adolescents, adults, and older adults.^3,5^ Recognizing the disease in adolescents and adults is important, considering that these age groups may be asymptomatic or oligosymptomatic carriers of *B. pertussis* and may transmit the pathogen to unimmunized or incompletely immunized newborns and children.^3,4,6–9^ Because of a progressive waning of immunity, pertussis could occur in adolescents and adults even when there is a history of complete immunization or natural disease in childhood.^3,10^ Despite the well-accepted definition of cases^1^ underreporting and misdiagnosis, particularly among adolescents and adults, is a problem worldwide.^12–14^ Adolescents and adults may present atypical symptoms limited to mild or prolonged non-distinctive cough, but they may also suffer severe symptoms such as sleep disturbance, pharyngeal symptoms, weight loss, sneezing attacks, sinus pain, sweating, and headaches.^2^

Still, data on the epidemiology and clinical characteristics of the disease in adolescent and adult populations are limited.^15,16^ Furthermore, findings suggest that pertussis incidence in adults aged >50 years old has been increasing over the past years^3^ but it remains significantly underestimated.^14^

We aimed to systematically review the epidemiology of pertussis disease in adolescents, adults, and older adults over the past decade in selected Latin American countries.

### Methods

Following the PRISMA guidelines, we systematically reviewed literature published between 2007 and 2018 reporting on pertussis epidemiological data from *in-scope* countries (Argentina, Brazil, Chile, Colombia, Mexico, Panama, and Uruguay). Countries were considered *in-scope countries* because they have incorporated in their national immunization program the diphtheria tetanus acellular pertussis (Tdap) vaccine for adolescents (Argentina, Panamá, Uruguay) and/or the Tdap vaccine as part of their maternal immunization strategy (Brazil, Panama, Argentina, Chile, Uruguay, Mexico and Colombia) (Supplementary Table 1).^4^

We aim to review the epidemiology of pertussis disease in adolescents, adults, and older adults (*in-scope* age groups) between 2007 and 2018 in the *in-scope countries* (Supplementary methods – Research question). For the age definition of the in-scope age groups, we used the definitions applied by the World Health Organization [WHO]:^5,6^ adolescents are aged 10–19 years, adults 20–59 years, and older adults ≥60 years.

## Eligibility criteria

Eligible studies were 1) primary studies, such as observational epidemiological studies, clinical trials, and systematic reviews of primary studies; 2) abstracts presented at scientific events and published in their respective proceedings; 3) studies published between 2007 and 2018; and 4) studies in English, Portuguese, or Spanish. Studies were excluded if their focus was not in the selected countries, or did not report epidemiological data (prevalence, incidence, hospitalizations, and mortality) of pertussis in adolescents, adults, and older adults.

### Information sources

The search was performed in June 2018 in the following databases: Medline, Embase, LILACS, SciELO, Google Scholar, CAPES Journals Web-portal, and Cochrane library.

### Study selection and data collection process

Identified studies were evaluated in two phases by two independent reviewers using the inclusion and exclusion criteria. In the first phase, the retrieved publications were screened for eligibility based on their titles and abstracts; the studies that passed this first screening stage progressed to the second phase, consisting of full-text content evaluation. Relevant information was extracted from all eligible articles by the two independent reviewers. Disagreement between the two researchers was eventually resolved by a third independent reviewer.

### Assessment of the risk of bias

The risk of bias in observational studies was assessed using Newcastle-Ottawa Scale (NOS) tool or its version adapted for cross-sectional studies.^7^ The clinical trials and systematic review publications were assessed by the Cochrane bias risk assessment tool and AMSTAR checklist, respectively.^8,9^

### Data extraction and analysis

Data reported with information from individuals >10 years old were considered relevant to our *in-scope* age group. The data collected included source data for each study, period of data collection, geographical region, cases definition, diagnostic tools, and the percent prevalence, incidence, and mortality due to pertussis in adolescents, adults, and older adults.

Primary measures were summarized and presented descriptively by outcome and country along with risk of bias and quality assessment. Due to considerable methodological differences in the study designs and reporting of outcomes among eligible studies, a meta-analysis was not conducted. All data were analyzed using *Excel* ™and *MedCalc* ™software.

## Results

### Systematic literature review

**Figure 1.**
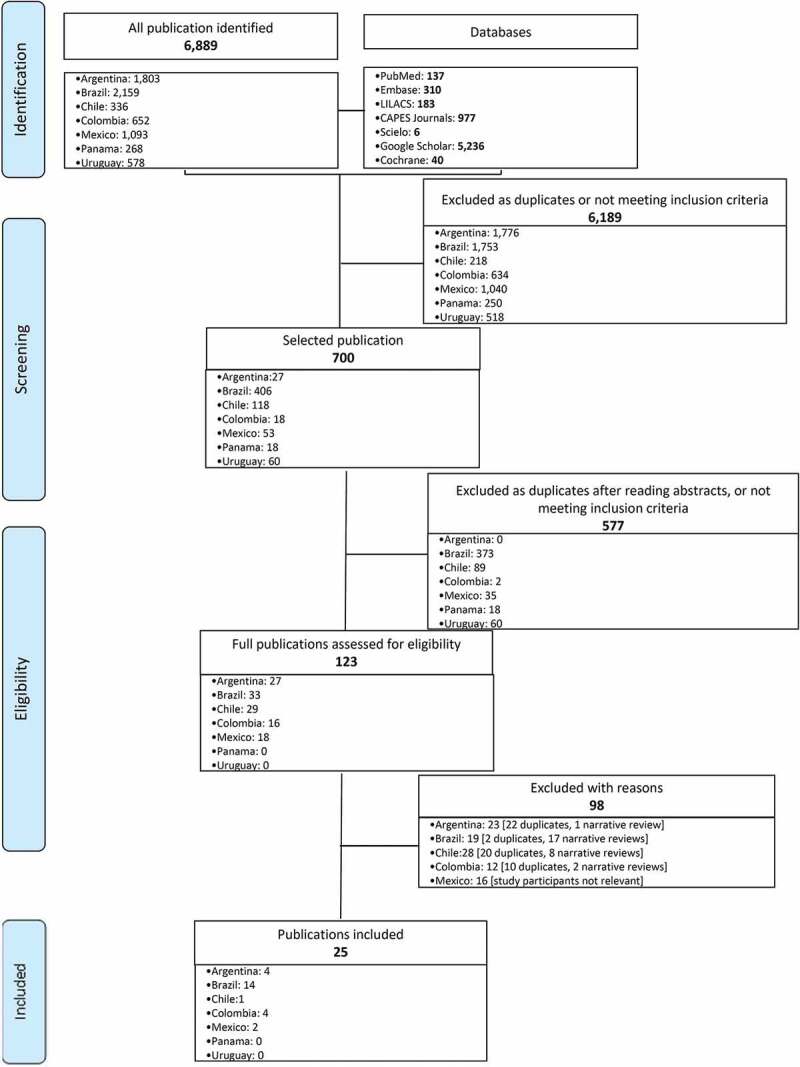
PRISMA Flow diagram of studies selection

Of the 6,891 references identified, most (89.8%, 6,189/6,891) were excluded at the first screening phase as duplicates (Figure 1). Of the 125 (18.0%) studies that made it to the second phase, only 25 were found eligible. Most studies (n = 14) were from Brazil, followed by Argentina and Colombia (n = 4 each), Mexico (n = 2), and Chile (n = 1). There were no eligible studies from Panama or Uruguay. Study design was predominantly cross-sectional, with only one case–control study, and one literature review. No clinical trial was included. Table 1 summarizes the characteristics and findings of these eligible studies.

**Table 1.** Summary of selected study characteristics, by country, and corresponding information on pertussis cases among adolescents, adults, and older adults

						Adolescents, adults, and older adults pertussis cases (% of all confirmed cases)		
Author, year^Ref^	Study period	Study design	Study’s data source	Study participants	Age group of interest*	overall	By age group**	Database†
***Argentina***								
***National level data***								
Hozbor *et al*., 2009^10^	2004–2007	Surveillance	SINAVE	Confirmed cases, all country	> 16 y	2.1%‡	n.r.	PubMed
Romanin *et al*., 2014^39^	2002–2011	Surveillance	SINAVE	All cases reported^§^, country	> 15 y	2.7%	n.r.	PubMed
***Regional level data***								
Kusznierz *et al*., 2014^20^	2006–2010	Cross-sectional	One tertiary hospital	Suspected for pertussis children < 14 y and the family contacts of confirmed cases, Santa Fe (city)	> 10 y	5.2%‡	n.r.	PubMed
Lavayén *et al*., 2017^21^	2011–2015	Cross-sectional	INE	Suspected cases, Mar del Plata (city)	> 7 y	5.7%‡	n.r.	Scopus
***Brazil***								
***National level data***								
Willemann *et al*., 2014^12^	2007–2011	Case-control	SINAN	*Cases*: confirmed pertussis cases *Controls*: notified as suspected cases, but were not confirmed as such	> 15 y	6.6%‡	n.r.	SciELO
Falleiros Arlant *et al*., 2014^23^	2011–2012	Cross-sectional	SINAN	Confirmed cases, all country	> 10 y	11.6%‡	*10–19y: 5.5%* *20–59y:* *5.8%* *≥60y:* 0.3%	Pubmed
Guimarães *et al*., 2015^28^	2007–2014	Surveillance	SINAN	All cases reported^§^, all country	> 15 y	8.8%‡	n.r.	Pubmed
Castro and Milagres, 2017^13^	2010-2014 2015	Surveillance	SINAN	Cases reported, whole country	> 15 y PW (3rd trimester)¶	15 y, 2010–2014: 10,1%‡	n.r.	LILACS
						PW, 2015: 167 cases¶		
Silva *et al*., 2017^26^	2001–2014	Cross-sectional	SINAN	All cases reported^§^, all country	> 10 y	13.1%‡ (all study period) 13.8%‡ (2007–2014)	*10–19y:* 5.9% *20–59y: 6.9%* *≥60y: 0.3%*	Google Scholar
***Regional level data***								
Druzian *et al*., 2014^22^	1999–2008	Cross-sectional	SINAN, LACEN	All cases reported^§^, Mato Grosso do Sul state	> 10 y	8.4%‡	n.r.	PubMed
Berezin *et al*., 2014^29^	2011–2012	Cross-sectional	Adolfo Lutz Institute	Household contacts of children with pertussis	> 10 y	7.9%¶	n.r.	PubMed
Bellettini *et al*., 2014^31^	2011–2013	Case series	One hospital clinic	Suspected cases, Santa Casa de Misericórdia Porto Alegre	> 10 y	9.3%‡	*10–19y: 8.1%* *40–59y:* *1.2%*	SciELO
Torres *et al*., 2015^25^	2007–2013	Cross-sectional	SINAN	Confirmed cases, Paraná State	> 10 y	13.4%‡	*10–19 y:* 5.2% *20–49y: 7.7%* *≥65 y:* 0.5%	PubMed
Cunegundes *et al*., 2015^30^	2011	Cross-sectional	Pediatric department of one tertiary hospital	Paediatric healthcare workers	> 21 y	6.4%¶	n.r.	PubMed
Pimentel *et al*., 2015^32^	2010–2011	Cross-sectional	10 outpatient clinics	Suspected cases	> 10 y	5.2%^¶^	n.r.	PubMed
Lima *et al*., 2016^11^	2009–2013	Cross-sectional	Population of Vitória municipality	Confirmed cases, Vitória da Conquista (city)	> 10 y	10.3%‡	*10–14 y*: 2.9% (I case) *20–39 y:7.7% (3* cases)	Google Scholar
Verçosa and Pereira, 2017 ^27^	2005–2015	Cross-sectional	SINAN	Confirmed cases, Alagoas state	> 10 y	14.0%‡	*10–19 y*: 9.0% *20–49 y:5.0%*	Google Scholar
Fernandes *et al*., 2018^28^	2001–2015	Surveillance	SINAN – CVE-SP	All cases reported^§^, São Paulo state	> 10 y	13.6%‡ (all study period) 13.7%‡ (2007–2015)	*10–20 y*: 4.3% *>20 y:9.3%*	PubMed
***Chile***								
***National level data***								
Lima *et al*., 2015^33^	1932–2010	Time series	National Epidemiological Surveillance System, Epidemiological Statistics Yearbooks	All cases reported^§^, all country	> 10 y	0.16–0.41 (per 100,000) (incidence rate for the period 2001–2010)	incidence rates per 100,000 for the period 2001–2010: *10–19 y*:0.41 *20–44 y*:0.30 *>45 y*:0.16	PubMed
***Colombia***								
***Regional level data***								
Villareal *et al*., 2008^15^	2008	Cross-sectional	Clínica Integral Sincelejo	Suspected cases between community living people, Sincelejo (city)	≥ 15 y	60%‡ (3 out of 5 cases)	*10–19 y*: 20% (I case) *20–59 y:40% (2* cases)	LILACS
Astudillo *et al*., 2011^36^	2006–2007	Cross-sectional	SIVIGILA for Cali	Suspected cases and household contacts of confirmed cases, Southeast Cali (city)	> 15 y	66.7%‡	*15–24 y*: 30.6% *25–64 y:33.3%*	Scopus
Ulloa-Virgüez, 2015^35^	2010–2012	Cross-sectional	SIVIGILA	All cases reported^§^, Antioquia, Nariño, Bogotá cases	>15 y	15.0%‡	*15–44 y*: 11.6% *>44 y:3.4%*	Scopus
Montilla-Escudero *et al*., 2016^34^	2013 (outbreak)	Cross-sectional	SIVIGILA	Suspected cases, Antioquia	> 10 y	15.7%‡	n.r.	Scopus
***Mexico***								
***National level data***								
Conde-Glez *et al*., 2014^37^	2010	Cross-sectional	National Institute of Public Health	National Health and Nutrition Survey, participants	> 10 y	40.4%–46.3%	n.r.	PubMed
Aquino-Andrade *et al*., 2017^38^	2011–2014	Cross-sectional	11 hospital clinics	Confirmed cases aged <1 y and their household contacts	> 16 y	19.6%	n.r.	PubMed

*only data corresponding to age groups >10 years old are presented, even if data for younger ages have been reported in the corresponding study; **preferably by the WHO age-group definitions [adolescents 10–19 years, adults 20–59 years, and older adults ≥60 years] or anything close to that;†the database from which the corresponding publication was retrieved; ‡(percentage) among confirmed pertussis cases; ^§^includes suspected and confirmed pertussis cases; ¶(percentage) among all participants (includes suspected, confirmed, and non-confirmed cases)

Abbreviations: CVE-SP. Centro de Vigilância Epidemiológica – São Paulo [São Paulo Satte Epidemiological Surveillance Center]; INE, Instituto Nacional de Epidemiología [(Bacteriology Service of the) National Institute of Epidemiology]; LACEN, Laboratório Central do Mato Grosso do Sul [Central Laboratory for Public Health]; n.r. not reported PW, pregnant women; SINAN, Sistema de Informação de Agravos de Notificação [System for Notifiable Diseases]; SINAVE, Sistema Nacional de Vigilância Epidemiológica [Argentinean National Epidemiological Surveillance System]; SIVIGILA, Sistema Nacional de Vigilancia en Salud Pública [National Public Health Surveillance System]; y, year(s)

### Argentina

Two studies presented national-level data,^21,22^ and two other regional-level data (Santa Fe and Mar del Plata cities).^23,24^

#### National-level data

The national-level studies used pertussis epidemiology data from the national surveillance system covering the period 2002–2011 and reported that among confirmed cases approximately 2.7% were >15 years-old. Between 2004 and 2007, one death due to pertussis was reported in the *in-scope* age group.^10^

#### Regional-level data

The reported frequency of pertussis in the two regional-level studies was twice as high as in the studies with national-level data. Kusznierz *et al*.^11^ study included household contacts of children with pertussis visiting a pediatric hospital in the city of Santa Fe, and the Lavayen *et al*.^24^ study included positive samples from private health services, primary care centers, municipal hospitals of Mar del Plata and one hospital in Buenos Aires province. In the former study,^11^ 102 (9.5%) of the 1,074 children <14 years-old with pertussis-like clinical characteristics were pertussis-confirmed cases and had 16 suspected for pertussis family contacts. All cases >10 years old were treated as outpatients. Lavayen *et al*. evaluated 572 pertussis-suspected cases.^24^ Using different methodologies (Table 2) the authors confirmed 88 of all suspected cases, five of which (5.7%) were >7 years-old.

**Table 2.** Definitions of pertussis cases, and diagnostic procedures reported in respective studies

Author, year^Ref^	Disease definition	Diagnostic procedure (% of confirmed cases*)
***Argentina***		
Hozbor *et al.*, 2009^10^	*Clinical case* Cough lasting ≥2 weeks with ≥1 of the symptoms: coughing paroxysms, inspiratory whoop, or post-tussive vomiting. *Confirmed case* a person with acute cough illness of any duration, and a positive culture for *B. pertussis* or a case that meets the clinical case definition and is confirmed by PCR, or a case that meets the clinical case definition and is confirmed by serology, if the last vaccine dose was received at least four years ago. A significant increase of overall antibody titer against *B. pertussis* whole cell (sonicated) in paired sera is used as criteria for serological confirmation or a case that meets the clinical case definition and is directly epidemiologically linked to a laboratory confirmed case.	n.r.
Kusznierz *et al.*, 2014^20^	*Convulsive cough* Cough of ≥7 days duration, paroxysmal or not, with inspiratory stridor or inducing vomiting. *Confirmed case* Clinically compatible patient with convulsive cough. *Index case* Patient with convulsive cough and confirmed pertussis by PCR. *Symptomatic contact* Patient with manifestations of convulsive cough. ‘Primary’ were if symptoms began ≥7 days before the index case, and ‘secondary’ if the onset of symptoms was at least 7 days after the index case. The primary cases were considered as the source of infection.	PCR (100%)
Romanin *et al*., 2014^39^	*Suspected case* Cough lasting ≥2 weeks with ≥1 of the symptoms: coughing paroxysms, inspiratory whoop, or post-tussive vomiting *Confirmed case* Suspected case with laboratory isolation of *B. pertussis*	Culture (n.r.) PCR (n.r.) Serological (n.r.)
Lavayén *et al.*, 2017^21^	<6 months. All acute respiratory infections with ≥1 symptom of: apnea, cyanosis, inspiratory stridor, vomiting after coughing or paroxysmal cough 6 months–11 years. Cough lasting ≥14 days, accompanied by ≥1 symptom of: paroxysmal cough, inspiratory stridor or vomiting after cough without other apparent cause >11 years. persistent cough lasting ≥14 days without other accompanying symptomatology	PCR (79%) PCR + Culture (19%) PCR + Culture + ELISA (2%)
***Brazil***		
Bellettini *et al.*, 2014^31^	n.r.	PCR (100%)
Berezin *et al.*, 2014^29^	n.r.	PCR (82.1%) Culture (7.1%) PCR + Culture (25.0%)
Druzian *et al.*, 2014^22^	*Suspected case* Dry cough lasting ≥2 weeks and ≥1 of: paroxysmal cough, inspiratory whoop, post-cough vomiting, having a history of contact with a pertussis case confirmed by clinical criteria. *Confirmed case* Laboratorial criteria: isolation of Bordetella pertussis Epidemiological criteria: suspected case which has had contact with a pertussis confirmed case by laboratory testing, between the beginning of the catarrhal period up to 3 weeks after onset of the paroxysmal period Clinical criteria: suspected case with leukocytosis (> 20,000 leukocytes/mm^3^) and absolute lymphocytosis (> 10,000 leukocytes/mm^3^) and negative or not performed culture; and absence of epidemiological linkage; and no confirmation of another etiology.	Laboratory criteria-Culture (7.6%) Epidemiological linkage (22.7%) Clinical criteria (68.1%)
Falleiros Arlant *et al*., 2014^23^	WHO-CDC definition	Culture (n.r.) PCR (n.r.)
Willemann *et al.*, 2014^12^	*Case* Cough and ≥1 of: paroxysmal cough, winch inspiratory or vomiting.	Laboratory criteria (n.r.) Epidemiological (n.r.) Clinical criteria (n.r.)
Cunegundes *et al.*, 2015^30^	n.r.	ELISA (positive if Bordetella pertussis-IgG > 62.5 IU/mL)
Guimarães *et al.*, 2015^24^	*Confirmed case* Laboratorial criteria: isolation of Bordetella pertussis using culture of PCR Epidemiological criteria: suspected case which has had contact with a pertussis confirmed case by laboratory testing, between the beginning of the catarrhal period up to 3 weeks after onset of the paroxysmal period Clinical criteria: suspected case with leukocytosis (> 20,000 leukocytes/mm^3^) and absolute lymphocytosis (> 10,000 leukocytes/mm^3^) and negative or not performed culture; and absence of epidemiological linkage; and no confirmation of another etiology.	Laboratory criteria (36.6%) Epidemiological + clinical (15.5%) Clinical criteria (47.2%)
Pimentel *et al.*, 2015^32^	*Confirmed case* Cough and a positive culture, or Clinical case and positive PCR. *Clinical case* Dry cough lasting ≥2 weeks and ≥1 of: paroxysmal cough, inspiratory whoop, post-cough vomiting Epidemiological criteria A clinical case with negative culture and/or PCR who had contact with a pertussis confirmed case by culture and/or PCR	Culture (10%) PCR (70%) Epidemiological linkage (30%)
Torres *et al*., 2015^25^	*Confirmed case* Clinical criteria: cough lasting ≥2 weeks and ≥1 of: paroxysmal cough, inspiratory, post-coughing vomiting Laboratory criteria: isolation of Bordetella pertussis using culture of PCR Epidemiological clinical criteria: suspected case which has had contact with a pertussis confirmed case by laboratory testing in the period of transmissibility.	Culture (n.r.) PCR (n.r.)
Lima *et al.*, 2016^11^	*Suspected case*^19,55^ Dry cough lasting ≥2 weeks and ≥1 of: paroxysmal cough, inspiratory whoop, post-cough vomiting; or having a history of contact with a pertussis case confirmed by clinical criteria. *Confirmed case* Laboratorial criteria: isolation of *Bordetella pertussis* Clinical and epidemiological criteria: suspected case which has had contact with a pertussis confirmed case by laboratory testing, between the beginning of the catarrhal period up to 3 weeks after onset of the paroxysmal period Clinical criteria: suspected case with leukocytosis (> 20,000 leukocytes/mm^3^) and absolute lymphocytosis (> 10,000 leukocytes/mm^3^) and negative or not performed culture; and absence of epidemiological linkage; and no confirmation of another etiology.	Laboratory criteria – Culture (n.r.) Epidemiological (n.r.) Clinical criteria (n.r.)
Castro and Milagres, 2017^13^	n.r.	n.r.
Silva *et al.*, 2017^26^	n.r.	n.r.
Verçosa and Pereira, 2017^27^	n.r.	Culture (n.r.) PCR (n.r.)
Fernandes *et al.*, 2018^28^	*Confirmed case* Clinical criteria: cough lasting ≥2 weeks and ≥2 of: paroxysmal cough, inspiratory, post-coughing vomiting Laboratory criteria: isolation of Bordetella pertussis using culture of PCR Clinical and epidemiological criteria: suspected case which has had contact with a pertussis confirmed case by laboratory testing in the period of transmissibility.	2007–2015 Culture / PCR (79%) Epidemiological criteria (21%)
***Chile***		
Lima *et al*., 2015^33^	*Suspected case* Cough ≥2 weeks and ≥1: paroxysms of coughing, inspiratory whoop, post-tussive vomiting without other apparent cause, or cough of shorter duration if the features of the clinical case are depicted. And in <6 months, respiratory infection that causes apnea. *Confirmed case* A suspected case that is laboratory confirmed or epidemiologically linked by the laboratory. *Compatible or clinical case* Suspected case which could not be demonstrated or confirmed by epidemiological association or confirmed at the laboratory.	n.r.
***Colombia***		
Villareal *et al.*, 2008^15^	*Probable cases* <3 months: presenting respiratory disease with repetitive episodes of paroxysmal cough or apneas accompanied or not of inspiratory stridor. 3 months–12 years: presenting respiratory disease with repeated episodes of sustained paroxysmal cough lasting ≥1 week or with repetitive episodes of intense paroxysmal cough accompanied by inspiratory stridor and cyanosis subsequent with any time of evolution. 12 years: presenting respiratory disease cough lasting ≥2 weeks accompanied or not by paroxysms, expectoration and vomiting. *Confirmed case* A probable case confirmed by PCR or DIF. *Confirmed case by epidemiological link* A probable case definition that is epidemiologically linked to a case confirmed by PCR or Direct Immunofluorescence.	Laboratory criteria (PCR or DIF) (1/5) Epidemiological linkage (4/5)
Astudillo *et al.*, 2011^36^	*Suspected case* Cough lasting ≥1 week and ≥1 symptom paroxysmal cough, inspiratory stridor, post-cough vomiting and no other apparent cause. *Atypical case* One of the following symptoms: respiratory infection picture without another symptom, paroxysm, coughing, prolonged cough followed by periods of apnea, cyanosis and inspiratory stridor.	PCR (33.8%) Culture (8.3%)
Ulloa-Virgüez., 2015^35^	n.r.	PCR (n.r.) DIF (n.r.)
Montilla-Escudero *et al*., 2016^34^	*Confirmed case* A probable case confirmed by Direct Immunofluorescence and subsequently verified by either PCR or ELISA.	PCR (n.a.†) DIF (n.a.†) ELISA (n.a.†)
***Mexico***		
Conde-Glez *et al.*, 2014^37^	*Positive cases* Anti-pertussis toxin antibody concentrations ≥45 FDA U/ml were considered to be seropositive according to the manufacturer’s.	ELISA (100%)
Aquino-Andrade *et al.*, 2017^38^	*Symptomatic contacts* Cough lasting ≥ 1 week during the last 3 weeks before onset of the diagnosed case’s symptoms, regardless of the presence of fever and rhinorrhea.	PCR (100%)
***WHO***^1^		
	*Clinical case* Cough lasting ≥2 weeks and ≥1 of: paroxysmal cough, inspiratory whoop, post-cough vomiting. *Laboratory criteria* Isolation of *B. pertussis* from a clinical specimen Positive PCR assay for pertussis Positive paired serology	
***CDC***^55^		
	*Clinical case* Cough lasting ≥2 weeks and ≥1 of: paroxysmal cough, inspiratory whoop, post-cough vomiting. *Laboratory criteria* Isolation of *B. pertussis* from a clinical specimen Positive PCR assay for pertussis *Epidemiologic linkage* Contact with a laboratory-confirmed case of pertussis	

*the percentage of confirmed cases by respective diagnostic method, if reported in the corresponding publication; †not applicable because the confirmed cases were tested by >1 method (DIF, PCR, and/or ELISA)

Abbreviations: CDC, Centers for Disease Control; DIF, Direct Immunofluorescence; ELISA, Enzyme-Linked Immunosorbent Assay; n.a., not applicable; n.r. not reported; PCR, Polymerase Chain Reaction; WHO, World Health Organization; FDA, Federal Drug Administration

#### Diagnostic procedures and disease definitions

Polymerase chain reaction (PCR) was introduced in Argentina for the diagnosis of pertussis in late 2004, while cultures were still considered to be the gold standard for pertussis diagnosis.^24^ Enzyme-Linked Immunosorbent Assay (ELISA) was also used in the Lavayen *et al*.,^10^ whilst cultures were still considered to be the reference method in the laboratory diagnosis of pertussis. Study for the serologic detection of *B. pertussis* antibodies (cutoff value of 93 UI/mL).

### Brazil

All 14 studies were published between 2014 and 2017 (Table 1). Nine of them^25–33^ included data from the national surveillance system SINAN (Sistema de Informação de Agravos de Notificação [System for Notifiable Diseases]); two used convenience samples of household contacts^34^ or healthcare workers;^35^ each used data from one^36^ or ten^37^ hospital clinics; and one^12^ study was a population-based study. We retrieved 11 cross-sectional studies,^25,26,28,29,31–35,37,38^ one case-control study,^13^ one case series,^3^^6^ and one literature review^14^ (Table 1). The pertussis-confirmed cases in the *in-scope* population reported by all included studies, ranged from 5.2% to 14.0% (Table 1).

Only the study by Guimarães *et al*.^28^ reported on complications and deaths (152 complications) in adolescents, adults, and older adults, with pneumonia being the most common with 83 (54.6%) cases reported. Two deaths were reported in the same age group, one due to pneumonia and one due to malnutrition.^28^ Among the confirmed cases there were 73 pregnant women.^28^

#### National-level data

One case-control^13^ and three cross-sectional^26,28,31^ studies reported data from across the country (Table 1). The case–control study^13^ had a large sample size of 16,078 cases including 1,278 cases >15 years old, strengthening the external validity of the reported pertussis frequency. However, the authors were concerned about the quality of the data and specifically, the way that pertussis cases were defined as the cases reported to SINAN did not specify the duration of the cough. The cross-sectional studies also used the SINAN data covering the period 2000–2014,^26,28,31^ and they reported proportions of adolescents, adults, and older adults that varied between 8.8% and 13.1% (Table 1).

#### Regional-level data

Nine studies^25,29,32–38^ reported regional-level data. Of these, one was the case-series study and the others were cross-sectional studies. Among the confirmed pertussis cases, the frequency of the disease among >10 years-old ranged from 5.2% to 14.0% (Table 1).

#### Diagnostic procedures and pertussis case definitions

Half of the studies used the definition of confirmed cases given by the Brazilian Ministry of Health (MoH) that includes the laboratory, epidemiological, and clinical criteria shown in Table 2. For the studies that did not describe the definitions used, it is reasonable to assume that those reporting SINAN data^30–32^ followed the MoH definitions because the surveillance guidance is standardized by the MoH. The most common diagnostic procedures were *B. pertussis* culture and PCR (introduced in routine diagnosis at the National reference Center in 2010,^15^ and in 2005 in the state of Paraná).^29^ Serological diagnosis using ELISA (cutoff value of 93 UI/mL) was used only in the study on healthcare workers (Table 2).^35^

### Chile

One national-level study,^40^ reporting 78 years of pertussis epidemiology, was identified (Table 1). The diagnostic methods used were not reported (Table 2). The authors presented a critical analysis of the epidemiological aspects of the evolution of the disease in the country over the period 1932 to 2010. Based on information from the National Epidemiological Surveillance System, for the period 2001–2010, the incidence rate was 0.41/100,000 in the age group 10–19 years-old, 0.3/100,000 in the age group 20–44 years old, and 0.16/100,000 for those > 45 years-old.^40^ The corresponding incidence rates during the previous decade (1991–2000) were 0.42/100,000, 0.21/100,000, and 0.07/100,000, showing that pertussis incidence increased among those >20 years old and especially in the older group of >45 years old.^40^ The authors attributed this increase to several factors, including the increased awareness of the disease with consequent improvements in disease diagnosis, and waning of immunity. The authors further reported a regional dynamic for the disease, given that the increases in pertussis incidence occurred only in the central and mainly southern regions and not in the northern parts of the country.^40^

### Colombia

All four studies included reported regional-level data and were cross-sectional in design. The reported frequencies of pertussis cases in the *in-scope* population ranged widely from 15% to more than 60% (Table 1).

The study of Villareal *et al*.^16^ was a field research conducted in 2008 among five family members, in a community affected by an outbreak in February of the same year. An active search was implemented in selected health units to identify additional cases. Probable cases were subjected to laboratory confirmation with either PCR or direct immunofluorescence (DIF).

Only five cases were confirmed in the study, three of which were in our *in-scope* age group. Because of the study design, selection bias was considered as a limiting factor for the generalizability of the results. Moreover, the authors mentioned that more than half of the probable cases in all age groups had not been reported and that departmental public health laboratories needed to expand their capacity to diagnose pertussis.^16^

A further study, Montilla-Escudero *et al*.,^17^ was conducted during the 2013 outbreak in the department of Antioquia and aimed to assess the correlation between the diagnostic techniques, DIF, PCR, and ELISA. To this end, an active community search for symptomatic contacts of patients with positive DIF results and an active institutional search consisting of a monthly review of surveillance data was conducted and resulted in the selection of 180 probable cases. Nasopharyngeal samples of these cases were analyzed in the public health laboratory of Antioquia, 74% were found positive using either PCR or ELISA, and nearly 16% were from >10 years old individuals (Table 1). The authors considered that their results were not robust enough for case confirmation due to the use of the DIF technique for the identification of probable cases and not cultures. They also expressed concern that although PCR is the technique recommended by the official guidelines for the diagnosis of pertussis, 33 of the 35 public health laboratories had not implemented the method, and that firma diagnosis could be provided only by the National Reference Laboratory, therefore compromising the surveillance capacity of the country in the event of an outbreak.^17^

Ulloa-Virgüez *et al*. also carried out research in the Department of Antioquia just before the 2013 outbreak.^18^ They reported that the year 2012 was marked by the highest incidence rate of pertussis in all age groups. They also reported that in the age group 15–44 years-old, the prevalence of the disease was 11.6%, while in those >44 years-old it was 3.4%.^18^ The authors commented that the apparent resurgence of the disease was in fact due to improvements in diagnosis and surveillance. They also suggested that there was a change in the epidemiology of the disease because of its appearance in adolescents and adults as a result of immunity waning after vaccination.^18^

In the study carried out in the city of Cali, Astudillo *et al*. focused on household contacts of suspected pertussis cases (n = 24).^19^ All infected children had a contact who was also infected, with few symptoms and limited resources to diagnose pertussis. However, using PCR, 33 (30.3%) of the 109 household contacts were found positive for pertussis, of which 22 (66.7%) were in our *in-scope* age group (25 to >65 years old).^19^

#### Diagnostic procedures and pertussis case definitions

Definitions of confirmed pertussis cases varied between studies due to type of diagnosis test performed (Table 2). PCR was reported in all studies as a diagnostic method, following DIF in two studies. Cultures and ELISA had also been used for diagnosis.

### Mexico

Of the two cross-sectional studies included, Conde-Glez *et al*.^45^ used samples obtained from the Nationa Health and Nutritional Survey, and Aquino-Andrade et al.^46^ used data from 11 hospitals, four in Mexico City and six across the country. The definition of a confirmed case and diagnostic methods differed between the two studies (Table 2). The pertussis-confirmed cases in the *in-scope* population ranged from 19.6% to 65.3% (Table 1).

Using ELISA in 3,984 study participants, Conde-Glez *et al*.^45^ developed a seroprevalence survey to measure the levels of anti-*B. pertussis* antibodies among different social strata. The participant’s ages ranged from one to 95 years old, and the seropositivity rates for anti-*B. pertussis* antibodies were 40.4% in the 10–19 years old, and 46.3% in the >20 years old. The authors observed a statistically significant difference in the levels of seroprevalence between children, adolescents and adults. They attributed the high presence of infection in adolescents and adults to waning of immunity, and in older adults to acute infection as these adults had most likely not received vaccination earlier in life.^45^

The focus in the study by Aquino-Andrade *et al*.^46^ was on contacts of infected children. Among the 434 contacts enrolled, 85 (19.6%) were found positive for pertussis using PCR. Among the positive contacts, the mothers had the highest positivity rates for *B. pertussis* (41 of 85, 48.2%) with a median age of 24 years (range 16–42 years). Among the 71 fathers, 12 (16.9%) were positive for *B. pertussis* and had a median age of 25 years (range 17–45 years). Of all contacts, 38.7% had an unknown result indicating possible colonization with *B. pertussis* and a potential source of transmission. Most of the positive contacts were symptomatic (77.6%).^46^

### Pertussis epidemiological data by age groups

In summary, among confirmed pertussis cases, the frequencies in the overall *in-scope* population aged >10 years ranged widely from 2.1% to 66.7% depending on country and sample characteristics (Table 1). Most studies did not report data by age group (Table 1). Only 11 out of the 25 studies included in the systematic review reported data that could be grouped into the three age categories of adolescent, adult, and older adult, although not always with the same cutoffs in the age-group definitions (Table 1). Based on these limited data, the lowest frequency of pertussis was observed in the older adult group, and highest in the adult group. Despite this, the discrepancies in population characteristics and the small sample sizes are a limitation on the age-group comparisons and conclusions.

### Risk of bias quality assessment

Risk of bias was performed for the full-text journal publications (n = 25) using the NOS tool for the 17 cross-sectional studies included and an adapted version for the other observational designs. The overall risk of bias of individual studies was considered low, with scores varying from 2 to 5 according to the instrument scale. The evidence found was considered limited and of low quality. In addition, the overall risk of bias was considered high due to a high design-specific source of bias which can be attributed to the nature of observational studies, specifically those using passive surveillance and laboratory data.

## Discussion

#### Summary of evidence

The present systematic review shows that there is a serious deficit of epidemiological data among adolescents, adults, and older adults in selected Latin American countries. Likewise, there are no studies that clearly assess the status of asymptomatic or oligosymptomatic *B. pertussis* carriers in these specific groups. Only 7 of the 25 papers reported national-level epidemiological data on pertussis. Even for Brazil, the country with the most publications retrieved, only four studies reported on national-level data related to the *in-scope* population. This reflects that pertussis surveillance in the review’s *in-scope* countries was possibly not focused on or suitable for the identification of these age groups.

Among all cases, the frequency of *B. pertussis* was different between countries and varied widely, among patients depending on country and sample characteristics. This observation applies to the overall in-scope population of individuals aged >10 years and also to the in-scope age-groups. Some studies that were based on secondary national surveillance databases reported lower proportion of pertussis cases among adolescents and adults than studies using regional data, as evidenced from studies in Argentina,^21–24^ Mexico,^46^ and Colombia.^17,18^ Additionally, studies investigating contacts of positive cases showed a high prevalence of pertussis isolation among people >15 years old.^44,46^ Also, Berezin et al.^34^ observed that mothers (11.8%) and fathers (12.9%) were the groups of adults with the highest positive rates among contacts of children positive for pertussis and highlighted that contacts with index cases can be positive for *B. pertussis* regardless of the presence of symptoms. In an outbreak investigation in Colombia, even without the laboratorial diagnosis, pertussis cases were confirmed based on the epidemiological link observed between confirmed cases among children and the adults, showing potential transmission across all ages.^16^ On the other hand, a seroprevalence survey developed in Mexico showed lower prevalence of anti-*B. pertussis* antibodies among adolescents (10–19 years old: 40.4%) and adults (≥20 years old: 46.3%) when compared to the children antibody levels (1–9 years old: 59.3%) indicating waning immunity among those older age groups.^45^

The definition of cases was not consistent in the selected studies. Brazilian studies followed the WHO definition for a clinical case (coughing lasting ≥2 weeks with ≥1 symptom of paroxysms, whooping, post-tussive vomiting) and laboratory confirmation (culture, PCR, serological).^1^ Although WHO does not recommend DIF for pertussis diagnosis, because it may give false positive or false-negative results,^20^ the method was used in two studies from Colombia^16,18^ as an initial indicator of a probable case. The diagnostic procedure used also varied from study to study. PCR is considered the most sensitive method for disease diagnosis and was used in most of the studies with 16 of 25 using this diagnostic approach, but it was not the only technique. Other studies reported using the older method of isolation from a culture, and ELISA. Culture is considered 100% specific for *B. pertussis* but it loses sensitivity after the second week of infection, increasing the risk of false-negative results,^21^ and it is not optimally sensitive in adolescents and adults.^4,48^ Nevertheless, culture was considered the 'gold standard' method in some laboratories^13,25,38^ and was used as a diagnostic method in many (n=11) of the studies included in this review. ELISA was also used as a diagnostic method in parallel to others^24,42^ or as the only diagnostic technique.^35,45^

#### Some evidence of pertussis in adults outside Latin America

Outside Latin America, underestimation of the disease incidence in populations other than children was also mentioned in publications. A prospective study was conducted in 12 European countries from 2007 to 2010 in 3,104 adults (≥18 years old) visiting a general practitioner due to acute cough lasting ≤28 days.^41^ On average, 3% were pertussis cases (from 0% to 6.2%, depending on the country).^41^ In a US study, in the absence of direct incidence estimates, Masseria et al.^17^ used inferential statistics to estimate the 2006–2010 incidence of pertussis among adults ≥50 years old with cough illness (ICD-9 codes for pertussis, pneumonia, cough, and acute bronchitis), using a private Practitioners’ claims database containing approximately 1 billion entries. The model suggested 2.5% of those 50–64 years old and 1.7% of those ≥65 years old were infected by *B. pertussis*; the respective average estimated incidences were 202 and 257 per 100,000. The annual incidence of cough illness due to pertussis was predicted to increase over the years, reaching 464 per 100,000 in 2010 (i.e., 94–264 times higher than the country reported incidence for people aged >40 years old). These estimates, depending on the year, were 42–105 times higher than medically confirmed pertussis cases.

In a recent systematic review on the burden of disease and vaccination status in adults >50 years old, Kandeil *et al*.^18^ identified only 44 epidemiological studies published worldwide between 2006 and 2016. Most studies were conducted in Europe (n = 18), followed by Australia and New Zealand (n = 10). Two studies were from South America, both from Brazil and are included in the present systematic review.^25^ Kandeil *et al*.^18^ study also made important discussion related to pertussis epidemiology in adults. The author mentioned that, although Torres *et al*.^25^ reported an increase in pertussis incidence from 2007 to 2013, it is not possible to infer that this increase affected the age group of people ≥50 years old because the annual incidence was not stratified by age. Kandeil *et al*.^18^ also noted that the frequency of <1% confirmed pertussis cases in patients ≥60 years old reported by Guimaraes *et al*.^22^ was highly uncertain due to 5 to 6-times rate of underreporting during the study period of 2007–2014.^18^ Overall, Kandeil *et al*.^18^ suggested that the real worldwide prevalence of pertussis in older adults (≥50 years old) is largely underestimated by up to several 1,000-fold, as indicated by seroprevalence data of the clinical and subclinical infections.

Underreporting of pertussis cases among older adults may occur for several reasons.^2^ Different diagnostic techniques have a direct impact on the number of cases reported. Bacterial culture is specific and sensitive in infants but it is not as sensitive as PCR in diagnosing pertussis in adolescents and adults.^19,35^ Pertussis in adults may have an atypical course,^35^ be asymptomatic, or present with milder symptoms than those occurring in children.^3^ A systematic review and meta-analysis on diagnostic accuracy and clinical characteristics of pertussis-associated symptoms showed that adults without paroxysmal cough or the presence of fever are very unlikely to have pertussis (low specificity for these symptoms).^1^ Whooping and post-tussive vomiting however indicate that the adult who presents with such symptoms constitutes a *suspected case* in need for further confirmation.^43^ Post-tussive vomiting which is a moderately sensitive and specific symptom in children was shown to have, together with whooping cough, a low sensitivity but a high specificity in adults.^43^ Furthermore, comorbidities in older adults such as chronic respiratory diseases make pertussis symptoms more difficult to detect, and cause delay or missed opportunities for disease diagnosis.^18,42,44^ A recent analysis of pertussis cases with cough onset between 2001 and 2015, found that 27% of patients ≥65 years old had a history of chronic obstructive pulmonary disease and 44% of those 12–20 years old had a history of asthma, both higher than the average rate in the US for the same age groups.^44^

Differences between vaccination policies might also have affected the epidemiological analysis by age group. Country-specific characteristics of immunization programs and time since their introduction should be investigated in future by examining disease prevalence and also vaccine uptake by age group. Evidence comparing the effects of vaccination policies on pertussis prevalence among our in-scope age-groups is not available. Furthermore, our study did not aim to investigate such effects, although we know from data on infants that there is such an association. For example, based on the 2017 Global Pertussis Initiative (GPI) report, the pertussis case fatality rate among infants was decreased in Latin America after the introduction of maternal immunization during pregnancy.^45^ Argentina was the first of the Latin America countries to introduce free universal maternal immunization during pregnancy in 2012, and in 2014, deaths due to pertussis were 92% lower than in 2011 {Vizzotti, 2015#180}. In Brazil too, pertussis immunization of pregnant women was associated with a 47.7% reduction in non-hospitalized pertussis cases among young infants aged <12 months after introduction of maternal vaccination in 2014.^46^

Expanding current knowledge on age groups neglected by current literature, which mostly involves hospitalized cases among infants and young children,^47^ our findings may be used to support efforts to extend vaccination policy in the countries in the region. This is primarily because they show that adolescent and adult pertussis epidemiology has been largely neglected in each in-scope country in terms of disease surveillance, diagnosis, and definition of cases. Acknowledging these shortcomings, the Pan American Health Organization (PAHO) established the Latin American Pertussis Project (LAPP) in 2009 with the aim of expanding diagnostic capacity and pertussis surveillance.^47^ Based on the available epidemiologic profile of the country in question, LAPP provides laboratory and epidemiologic training, and technical assistance.^47^ Evaluation of vaccine policies is the next step followed by surveillance with valuable inputs from sharing of each country’s experiences and best practices.^47^ With the same aim, the 1st Regional Experts on Infant Vaccination (REIV) meeting in 2018 in Colombia concluded that epidemiological data in the area must be updated, and include risk groups; case definitions should also be harmonized among countries in the region.^27^ Furthermore, vaccination strategies should strengthen and include missed opportunities such as visits not related to preventive care.^48–50^ Published findings show that adolescents up-to-date with other vaccinations are more likely to receive an additional age-appropriate vaccine. Thus, simultaneous vaccination is an effective measure to increase vaccine uptake and shows the usefulness of adolescent and adult vaccination platforms.^51–53^

#### Study limitations

Many of the selected studies had only a limited description of the epidemiological profile of pertussis among adolescents, adults, and older adults. However, the results to the least suggest the disease prevalence among adolescents and adults is sufficient to perpetuate the circulation of *B. pertussis*, as they serve as a reservoir for the pathogen, thus placing young children at permanent risk of developing pertussis and suffering the consequences of the disease. The findings of the present review are limited in terms of the absence of age-specific definitions for the disease, the possible underestimation and underreporting of cases, and the variations between countries with regards to disease definition and diagnostic procedures. Standardized methods for disease definitions by age group, and for sample collection, transportation, and diagnostic procedures are mandatory if comparable data within and across the Latin America region are to be produced.^54,56^ Finally, we considered studies from only seven Latin America countries, so our findings cannot be generalized to the Latin America region.

### Conclusions

Existing pertussis epidemiological data for adolescents, adults, and older adults of Latin American countries could underestimate the real burden of disease in these populations. Reliable information is required on disease prevalence in a population that is frequently asymptomatic and is a major source of pathogen transmission to children. Our findings showed a need to standardize and apply robust diagnostic tools and have appropriate surveillance systems on a national level. Epidemiological data might be underestimated by the current surveillance methods used by the study countries, such that estimates of prevalence in adolescents and adults may not be reliable enough to serve as a basis for evidence-based policies.

Considering the gaps in knowledge about the disease occurrence among other age groups, preventive strategies such as vaccination among pregnant women, adolescents, adults, and healthcare professionals working in childcare become essential to control the disease transmission among the most vulnerable populations (newborns and children). In the countries where the vaccination with the acellular anti-pertussis vaccine is already implemented for pregnant women, adolescents, and other specific population, an uptake on the vaccination coverage is crucial to avoid the infection to the children.

Additionally, the health authorities should develop and implement improved and age-specific methods for disease diagnosis, in addition of offering appropriate training to physicians who are responsible for disease recognition.

Also, the health authorities should take appropriate action to increase awareness about adolescent and adult pertussis infection in the public and encourage physicians to include the disease in their differential diagnosis.

Finally, there is a clear need for well-designed large-scale observational studies on the epidemiology of pertussis in Latin American countries.

## Supplementary Material

Supplemental Material
