# Supplementary material for: Epidemiology of pertussis among adolescents, adults, and older adults in selected countries of Latin American: a systematic review
Source: Hum Vaccin Immunother. 2021 Mar 18;17(6):1733–46. doi: 10.1080/21645515.2020.1827613 (PMC8115456; doi:10.1080/21645515.2020.1827613)
Supplement: Supplemental Material [file KHVI_A_1827613_SM4986.docx]

**Supplementary Table 1.** Summary of pertussis immunization schedules by country and available vaccine types

|  | **First year** | **1st booster** | **2nd booster** | **Adolescents (year*)** | **PW (year*)** | **Postpartum** | **HCP** |
| --- | --- | --- | --- | --- | --- | --- | --- |
|  | **2, 4, 6 m  Vaccine type** | **15–18 m** | **4–6 y** |  |  |  |  |
| Argentina | DTP-Hib-HB | DTP-Hib | DTP | Tdap (2009) | Tdap (2012) | Tdap | Tdap |
| Brazil | DTP-Hib-HB | DTP | DTP | No | Tdap (2014) | Yes | Tdap |
| Chile | DTP-Hib-HB | DTP-Hib-HB | Tdap | Tdap (2012) | Tdap (2017) | No | No |
| Colombia | DTP-Hib-HB | DTP | DTP | No | Tdap (2013) | No | No |
| Mexico | Tdap-Hib-IPV | Tdap-Hib-IPV | DTP | Tdap (2012) | Tdap (2012) | No | No |
| Panama | DTP-Hib-HB | DTP-Hib | DTP | Tdap (2014) | Tdap (2012) | Tdap | Tdap |
| Uruguay | DTP-Hib-HB | DTP-Hib-HB | DTP | Tdap (2012) | Tdap (2015) | Tdap | Tdap |

*year of introduction

Sources: (1) Organización Panamericana de la Salud (OPS). Inmunización: Datos, mapas y estadísticas <https://www.paho.org/hq/index.php?option=com_topics&view=rdmore&cid=7342&Itemid=40929&lang=es> ; (2) Falleiros Arlant L et al. (2014). Pertussis in Latin America: epidemiology and control strategies. *Expert. Rev. Anti. Infect. Ther* **12**, 1265-1275

Abbreviations: DTP, diphtheria-tetanus-pertussis; HB, hepatitis B; HCP, health care provider; Hib, *Haemophilus influenzae (H. influenza)* type B; IPV, inactivated polio vaccine; m, month(s); PW, pregnant women; Tdap, diphtheria tetanus acellular pertussis; y, year(s)
